# Estimation of treatment preference effects in clinical trials when some participants are indifferent to treatment choice

**DOI:** 10.1186/s12874-017-0304-x

**Published:** 2017-02-20

**Authors:** Stephen D. Walter, Robin M. Turner, Petra Macaskill, Kirsten J. McCaffery, Les Irwig

**Affiliations:** 1 Department of Clinical Epidemiology and Biostatistics, McMaster University, CRL 233, Hamilton, ON Canada L8N 3Z5; 2 School of Public Health and Community Medicine, University of New South Wales, Sydney,, NSW 2052 Australia; 3 Screening and Test Evaluation Program, Sydney School of Public Health, Sydney Medical School, University of Sydney, Sydney, NSW 2006 Australia

**Keywords:** Randomised trials, Design, Estimation, Participant preferences

## Abstract

**Background:**

In the two-stage randomised trial design, a randomly sampled subset of study participants are permitted to choose their own treatment, while the remaining participants are randomised to treatment in the usual way. Appropriate analysis of the data from both arms of the study allows investigators to estimate the impact on study outcomes of treatment preferences that patients may have, in addition to evaluating the usual direct effect of treatment. In earlier work, we showed how to optimise this design by making a suitable choice of the proportion of participants who should be assigned to the choice arm of the trial. However, we ignored the possibility of some participants being indifferent to the treatments under study. In this paper, we extend our earlier work to consider the analysis of two-stage randomised trials when some participants have no treatment preference, even if they are assigned to the choice arm and allowed to choose.

**Methods:**

We compare alternative characterisations of the response profiles of the indifferent or undecided participants, and derive estimates of the treatment and preference effects on study outcomes. We also present corresponding test statistics for these parameters. The methods are illustrated with data from a clinical trial contrasting medical and surgical interventions.

**Results:**

Expressions are obtained to estimate and test the impact of treatment choices on study outcomes, as well as the impact of the actual treatment received. Contrasts are defined between patients with stated treatment preferences and those with no preference. Alternative assumptions concerning the outcomes of undecided participants are described, and an approach leading to unbiased estimation and testing is identified.

**Conclusions:**

Use of the two-stage design can provide important insights into determinants of study outcomes that are not identifiable with other designs. The design can remain attractive even in the presence of participants with no stated treatment preference.

**Electronic supplementary material:**

The online version of this article (doi:10.1186/s12874-017-0304-x) contains supplementary material, which is available to authorized users.

## Background

Clinical trial outcomes may be affected by preferences that trial participants might have between the treatments under comparison. Preference effects can be substantial [1], but they are unobservable in standard trial designs; however, they are estimable in the two-stage randomised trial, in addition to the usual treatment effect [2, 3]. Using this design, a randomly sampled subset of patients are permitted to choose their own treatment, with the remainder randomised to treatment in the usual way. Estimated preference effects often provide additional insight into determinants of the study response, beyond that of the treatment effect alone [1–4]. As we have reviewed elsewhere [1], several preference-based designs have been employed in biomedical research in recent years [5–18].

We previously showed how to optimise the two-stage design by controlling the proportion of patients who are allocated to the choice arm (and hence can choose their treatment) versus the random arm (where they are randomised to treatment) [3]. In our previous illustrative example, involving the management of women with abnormal cervical screening results [4, 5], the investigators compared a novel intervention with standard care, which effectively defined a treatment preference for all participants. However, in other situations, some participants may have no treatment preference, despite being informed about the alternatives. For instance, in our further example discussed later [6, 7], 69% of participants in the choice arm had no preferred treatment.

In general, patients may be unable or unwilling to choose a treatment because they want the doctor to decide, or through anxiety or a lack of information or decision-making confidence. Note specifically that “no preference” is the *observed* preference value obtained after due process of presenting treatment options to the patient, but it is not “missing” data.

No previous paper has discussed how to deal with patients who are indifferent to treatment choice, i.e., who are “undecided”, in more than passing fashion. Apart from our own previous work [1], parameter estimation in the two-stage design has not been described. The possibility of undecided patients was mentioned when the design was originally proposed by Rucker [2], but only concerning significance testing. Rucker assumed that such participants either did not exist, or that preference effects did not apply to them. We show here that these approaches result in potentially biased parameter estimates, but also that alternative (and testable) assumptions are possible. We believe this issue is an important gap in the existing literature, on a topic with considerable practical applicability.

Our previous work assumed that all patients had a treatment preference, but we now extend our methods to allow for undecided patients. We obtain estimates, variances and test statistics for various preference effects, and estimate the usual treatment effect in this design. We provide a general characterisation of the undecided group, including alternative assumptions about their response profile, and we determine if these assumptions are empirically testable.

We illustrate the calculations using actual trial data, discuss alternative ways that data from undecided participants can be used, and compare our analysis to that of the original trial investigators. In our Discussion, we consider the appeal of the two-stage design when participants with no treatment preference occur, including their impact on study feasibility and efficiency.

## Methods

**Fig. 1:**
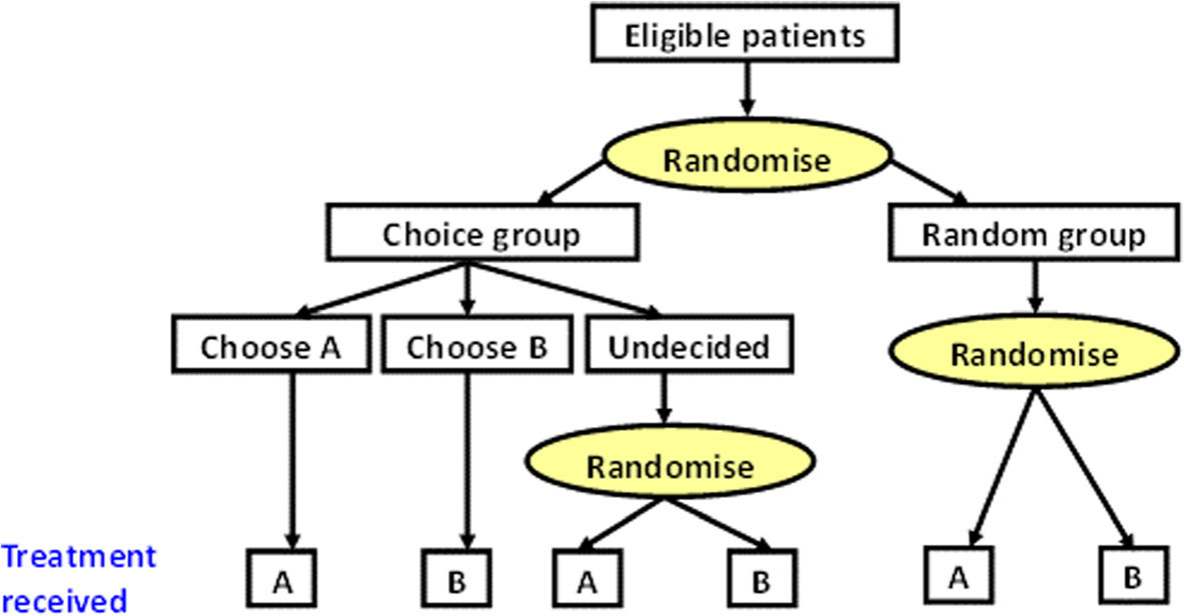
The two-stage randomised trial design

In the two-stage randomised trial design, participants are first randomly divided between the random and choice arms (see Fig. 1). In the random arm, participants are randomised to treatments A or B in the usual way. In the choice arm, participants are allowed to choose their treatment if they have a preference, while participants with no preference are randomised.

Following Rucker [2], we adopt the model:

1 $$ {Y}_{i j k}=\mu +{\tau}_i+{\nu}_j+{\pi}_{i j}+{\varepsilon}_{i j k} $$

where *Y*_*ijk*_ is the study outcome for participant *k* who receives treatment *i* (*i* = 1 for treatment A, 2 for treatment B) and who is in treatment preference group *j* (*j* = 1 for participants preferring A, *j* = 2 for participants preferring B, and *j* = 3 for participants with no preference). The parameters *τ*, *ν*, and *π* characterise the treatment, selection and preferences effects, as defined below; *ε* is a random error term, assumed independent of the other terms. As previously, we modify Rucker’s notation by replacing *σ* with *ν*, to avoid confusion between preference effects and standard deviations that are required later.

To eliminate parameter redundancies, we add four sets of constraints:

$$ {\tau}_1+{\tau}_2=0, $$

$$ \alpha {\nu}_1+\beta {\nu}_2+\gamma {\nu}_3=0, $$

$$ \alpha {\pi}_{i1}+\beta {\pi}_{i2}+\gamma {\pi}_{i3}=0, f o r\  i = 1,\ 2 $$

and

$$ {\pi}_{1 j}+{\pi}_{2 j}=0\  f o r\  j = 1,\ 2,\ 3. $$

where *α, β* and *γ* are the expected proportions of participants in the choice arm who choose treatment A, treatment B, or make no choice, respectively.

Table 1 gives our notation. To simplify the expressions and their interpretation for estimates and test statistics to be derived later, we retain *Y* as the response variable in the random arm, but use *X* for the response in participants with a treatment preference in the choice arm, again following the original formulation [2]. We also denote *V* as the outcome among undecided participants in the choice arm. The total sample size is *N*, with *m* and *n* participants being allocated to the choice and random arms. For simplicity we assume equal treatment group sizes in the random arm.

**Table 1:** Observations and parameters by group and actual treatment for the 2-stage randomised design

Actual treatment		Choice arm (Total sample size = m)			Random arm (Total sample size = n)
		Choose A	Choose B	Undecided (and randomised)	
A	Sample size	*m*_*1*_	—	*m*_*3*_ */2*	*n*_*1*_
	Observations	*x*_*11*_ *,…x*_*1,m1*_		*v*_*11*_ *,…v*_*1,m3/2*_	*y*_*11*_ *,… y*_*1,n1*_
	Mean	*μ*_*11*_	*(μ*_*12*_ *)*	*μ*_*13*_	*μ*_*1*_
	SD	*σ*_11_		*σ*_13_	*σ*_1_
B	Sample size	*—*	*m*_*2*_	*m*_*3*_ */2*	*n*_*2*_
	Observation		*x*_*21*_ *,…x*_*2,m2*_	*v*_*21*_ *,…v*_*2,m3/2*_	*y*_*21*_ *,…y*_*2,n2*_
	Mean	*(μ*_*21*_ *)*	*μ*_*22*_	*μ*_*23*_	*μ*_*2*_
	SD		*σ*_22_	*σ*_23_	*σ*_2_

μ_12_ and μ_21_ are not observable

Ideally, we would like to observe ${\mu}_{ij}$ as the mean response for participants in preference group *j* who receive treatment *i*, for every (*i, j*) combination, but only four of the six possible subgroups are observable*.* We can observe participants in the choice arm who have a treatment preference, with expected outcomes ${\mu}_{11}$ and ${\mu}_{22}$ for participants who prefer and receive treatments A or B, respectively. Because undecided participants are re-randomised, we can estimate ${\mu}_{13}$ and ${\mu}_{23}$ from the choice arm. The remaining two subgroups, corresponding to ${\mu}_{12}$ and ${\mu}_{21}$, are unobservable, because all participants in the choice arm with a treatment preference will receive that treatment.

Among the *m* participants in the choice group, there are *m*_*1*_ and *m*_*2*_ who choose treatments A and B, respectively. Among the remaining participants with no preference, we assume that *m*_*3*_/2 are randomised to each of A and B. In the random arm, there is only one observed group for each treatment, containing unknown mixtures of treatment preferences. Here we observe samples of size *n*_*1*_ and *n*_*2*_ under treatments A and B.

Because of the potential effects of treatment preferences, we take a general approach and allow for different expected outcomes for the corresponding treatment groups in the choice and random arms, and for indifferent participants to have different expected outcomes from the other observable groups. We comment later on the implications of alternative assumptions concerning the responses in the indifferent participants.

We define the direct treatment effect by *Δτ* = *τ*_1_ − *τ*_2_, or equivalently *μ*_1_ − *μ*_2_. One can estimate *Δτ* from the random arm, through the sample estimate ${\overline{Y}}_1$ – ${\overline{Y}}_2$ of *μ*_*1*_ *– μ*_*2*._ By virtue of the randomisation, this estimate will be unbiased, because participant preferences will be balanced in expectation. However, the estimate will be less precise than in a parallel group trial of the same total size.

We now consider the selection and preference effects. Because there are three preference groups, there are two possible contrasts for each effect.

### Selection effect

The first selection effect is defined *by* Δ*ν = ν*_*1*_ *– ν*_*2*_ *,* being the expected difference in responses between participants who would choose treatment A versus treatment B, if allowed to do so. Equivalently

2 $$ \varDelta \nu =\left[\left({\mu}_{11}+{\mu}_{21}\right)-\left({\mu}_{12}+{\mu}_{22}\right)\right]/2, $$

which involves two unobservable means, *μ*_12_ and *μ*_21_. The first stage randomisation implies equal expected distributions of preferences in the choice and random arms. Hence, the mean for the random arm under treatment A is a weighted average of the means in the three preference groups; thus

$$ {\mu}_1=\alpha {\mu}_{11}+\beta {\mu}_{12}+\gamma {\mu}_{13} $$

from which the unobservable *μ*_12_ can be expressed in terms of observable quantities, as

$$ {\mu}_{12}=\left[{\mu}_1-\alpha {\mu}_{11}-\gamma {\mu}_{13}\right]/\beta, $$

and similarly for *μ*_21_ *.* From (1) we can show that *ν*_*j*_ = ½ [*μ*_*1j*_ *+ μ*_*2j*_] – *μ*, for *j* = 1, 2. By substituting for the two unobservable means in (2), we derive Δ*ν* in terms of observables, as:

3 $$ \varDelta \nu =\frac{1}{2}\left[{\mu}_{11}\left(1+\frac{\alpha}{\beta}\right)-\frac{\mu_1}{\beta}+\frac{\mu_2}{\alpha}-{\mu}_{22}\left(1+\frac{\beta}{\alpha}\right)-\frac{\mu_{23}\gamma}{\alpha}+\frac{\mu_{13}\gamma}{\beta}\right] $$

To estimate Δ*ν*, we may substitute into (3) the six observable sample means, and empirical estimates of the preference proportions from the choice arm, $\widehat{\alpha}={m}_1/ m$, $\widehat{\beta}={m}_2/ m$, and $\widehat{\gamma}={m}_3/ m$. After simplification, we obtain

4 $$ \overset{\wedge }{\varDelta \nu}=\left[\left({z}_1-{z}_2\right)-\widehat{\gamma}\left({w}_1-{w}_2\right)\right]/\left[2\widehat{\alpha}\widehat{\beta} m\right] $$

where ${z}_1={m}_1\left({\overline{X}}_1-{\overline{Y}}_1\right)$, ${z}_2={m}_2\left({\overline{X}}_2-{\overline{Y}}_2\right)$, ${w}_1={m}_1\left({\overline{X}}_1-{\overline{V}}_1\right)$ and ${w}_2={m}_2\left({\overline{X}}_1-{\overline{V}}_2\right)$. If there are no indifferent participants (*γ* = 0, and hence *β* = 1 – *α*), result (4) simplifies to the same estimator as in our previous work ([3], Eq. 2).

### Preference effect

The preference effect can be regarded as the interaction between a participant’s preferred treatment and the treatment actually received, or equivalently the difference between the treatment effects (A - B) for participants who would have chosen A or B. It can be defined as

$$ \varDelta \pi =\left({\pi}_{11}-{\pi}_{12}-{\pi}_{21}+{\pi}_{22}\right)/2 $$

or in terms of the mean outcomes

$$ \varDelta \pi =\left[\left({\mu}_{11}-{\mu}_{21}\right)-\left({\mu}_{12}-{\mu}_{22}\right)\right]/2 $$

This involves the unobservables *μ*_12_ and *μ*_21_, but if we again invoke the equivalence of the preference distributions in the choice and random arms, we obtain

$$ \varDelta \pi =\frac{1}{2}\left[{\mu}_{11}\left(1+\frac{\alpha}{\beta}\right)-\frac{\mu_1}{\beta}-\frac{\mu_2}{\alpha}+{\mu}_{22}\left(1+\frac{\beta}{\alpha}\right)+\frac{\mu_{13}\gamma}{\beta}+\frac{\mu_{23}\gamma}{\alpha}\right] $$

which involves only estimable quantities. Similarly to the derivation for the selection effect, we estimate the preference effect as:

5 $$ \overset{\wedge }{\varDelta \pi}=\left[\left({z}_1+{z}_2\right)-\widehat{\gamma}\left({w}_1+{w}_2\right)\right]/\left[2\widehat{\alpha}\widehat{\beta} m\right] $$

Again, this devolves to a simpler previous result when there are no undecided participants ([3], Eq. 3).

### Second contrasts for selection and preference effects

For the selection effect, we can define a second contrast as:

$$ \varDelta {\nu}^{\hbox{'}}={\nu}_3-\frac{\nu_1+{\nu}_2}{2} $$

which represents the difference in outcomes between participants with no preference vs. those with a definite treatment preference. This is orthogonal to Δ*ν*, and can be expressed as

$$ \varDelta {\nu}^{\hbox{'}}=\left[\left({\mu}_{13}+{\mu}_{23}\right)-\frac{1}{2}\left({\mu}_{11}+{\mu}_{21}+{\mu}_{12}+{\mu}_{22}\right)\right]/2 $$

which is equivalent to

$$ \varDelta {\nu}^{\hbox{'}}=\frac{1}{4\alpha \beta}\left[\alpha \left({\mu}_{11}-{\mu}_1\right)+\beta \left({\mu}_{22}-{\mu}_2\right)-2\alpha \beta \left({\mu}_{11}+{\mu}_{22}-{\mu}_{13}-{\mu}_{23}\right)-\gamma \left\{\alpha \left({\mu}_{11}-{\mu}_{13}\right)+\beta \left({\mu}_{22}-{\mu}_{23}\right)\right\}\right] $$

when expressed in terms of estimable quantities. By again substituting empirical sample means and preference proportions, we have an estimator

$$ \overset{\wedge }{\varDelta {\nu}^{\hbox{'}}}=\left[\left({z}_1+{z}_2\right)-\left({w}_1+{w}_2\right)+\left(\widehat{\alpha}-\widehat{\beta}\right)\left({w}_1-{w}_2\right)\right]/\left[4\widehat{\alpha}\widehat{\beta} m\right]. $$

Similarly, a suitable second contrast for the preference effect is

$$ \varDelta {\pi}^{\hbox{'}}=\left[\left(\frac{\pi_{11}+{\pi}_{12}}{2}-{\pi}_{13}\right)-\left(\frac{\pi_{21}+{\pi}_{22}}{2}-{\pi}_{23}\right)\right]/2, $$

which represents the difference in treatment effects (A vs. B) between participants with no preference vs. those with any definite preference. It is orthogonal to *Δπ*, and in terms of the observable means is

$$ \varDelta {\pi}^{\hbox{'}}=\frac{1}{4\alpha \beta}\left[\alpha \left({\mu}_1-{\mu}_{11}\right)-\beta \left({\mu}_2-{\mu}_{22}\right)+2\alpha \beta \left({\mu}_{11}-{\mu}_{22}-{\mu}_{13}+{\mu}_{23}\right)+\gamma \left\{\alpha \left({\mu}_{11}-{\mu}_{13}\right)-\beta \left({\mu}_{22}-{\mu}_{23}\right)\right\}\right] $$

with a corresponding estimator

$$ \overset{\wedge }{\varDelta {\pi}^{\hbox{'}}}=\left[-\left({z}_1-{z}_2\right)+\left({w}_1-{w}_2\right)-\left(\widehat{\alpha}-\widehat{\beta}\right)\left({w}_1+{w}_2\right)\right]/\left[4\widehat{\alpha}\widehat{\beta} m\right] $$

### Significance tests and confidence intervals for selection and preference effects

To test the null hypothesis of no selection effect (H0: Δ*ν* = 0), we can use the inner component of (4) as a test statistic, i.e.,

6 $$ T=\left({z}_1-{z}_2\right)-\widehat{\gamma}\left({w}_1-{w}_2\right) $$

An approximately normal z-statistic is given by $z= T/ v a r(T)$, where

7 $$ \begin{array}{l}\operatorname{var}(T)= m\Big\{\alpha {d}_1^2+\beta {d}_2^2-{\left(\alpha {d}_1-\beta {d}_2\right)}^2+{\left(1-\gamma \right)}^2\left(\alpha {\sigma}_{11}^2+\beta {\sigma}_{22}^2\right)+2\left(\frac{\theta}{1-\theta}\right)\left({\alpha}^2{\sigma}_1^2+{\beta}^2{\sigma}_2^2\right)\\ {}\kern4em +2\gamma \left({\alpha}^2{\sigma}_{13}^2+{\beta}^2{\sigma}_{23}^2\right)+\gamma \left(1-4\gamma \right){\left(\alpha {e}_1-\beta {e}_2\right)}^2+{\gamma}^2\left(\alpha {e}_1^2+\beta {e}_2^2\right)\\ {}\kern4.25em -2\gamma \left[\alpha \left(1-2\alpha \right){d}_1{e}_1+\beta \left(1-2\beta \right){d}_2{e}_2+2\alpha \beta \left({d}_1{e}_2-{e}_1{d}_2\right)\right]\Big\}\end{array} $$

with *d*_1_ = *μ*_11_ − *μ*_1_ *, d*_2_ = *μ*_22_ − *μ*_2_ *, e*_1_ = *μ*_11_ − *μ*_13_ *, e*_2_ = *μ*_22_ − *μ*_23_ *, θ* = *m*/*N* (the proportion of participants assigned to the choice arm), and where we have assumed for simplicity *n*_*1*_ = *n*_*2*_ = *N*/2. An outline of this and later derivations is given in the Additional file 1.

Because $\overset{\wedge }{\varDelta \nu}= T/\left[2\widehat{\alpha}\widehat{\beta} m\right]$ *,* further development gives

8 $$ \operatorname{var}\Big(\overset{\wedge }{\varDelta \nu \Big) \approx}\left[\operatorname{var}(T)+\frac{T^2}{\alpha^2{\beta}^2}\operatorname{var}\left(\widehat{\alpha}\widehat{\beta}\right)-\frac{2 T}{\alpha \beta}\operatorname{cov}\left( T,\widehat{\alpha}\widehat{\beta}\right)\right]/{\left[2\alpha \beta m\right]}^2 $$

where *cov* denotes a covariance. This leads to

9 $$ \begin{array}{l}\operatorname{var}\left(\overset{\wedge }{\varDelta \nu \Big)\approx }\ \right[\operatorname{var}(T)+\frac{T^2}{m\alpha \beta}\left(\alpha +\beta -4\alpha \beta \right)\\ {}\kern3.75em -\frac{2 T\gamma}{\alpha \beta}\left\{\alpha \left(1-2\alpha \right){d}_1-\beta \left(1-2\beta \right){d}_2-\alpha \beta \left[\left(1-4\alpha \right){e}_1-\left(1-4\beta \right){e}_2\right]\right\}\Big]/{\left[2\alpha \beta m\right]}^2\end{array} $$

We can use variance (9) to establish a confidence interval for *Δν*.

Similar results emerge for the preference effect *Δπ*. For testing the null hypothesis of no preference effect (H0: *Δπ* = 0) we use

$$ T*=\left({z}_1+{z}_2\right)-\widehat{\gamma}\left({w}_1+{w}_2\right) $$

with

10 $$ \begin{array}{l}\operatorname{var}\left( T*\right)= m\Big\{\alpha {d}_1^2+\beta {d}_2^2-{\left(\alpha {d}_1+\beta {d}_2\right)}^2+{\left(1-\gamma \right)}^2\left(\alpha {\sigma}_{11}^2+\beta {\sigma}_{22}^2\right)+2\left(\frac{\theta}{1-\theta}\right)\left({\alpha}^2{\sigma}_1^2+{\beta}^2{\sigma}_2^2\right)\\ {}\kern4em +2\gamma \left({\alpha}^2{\sigma}_{13}^2+{\beta}^2{\sigma}_{23}^2\right)+\gamma \left(1-4\gamma \right){\left(\alpha {e}_1+\beta {e}_2\right)}^2+{\gamma}^2\left(\alpha {e}_1^2+\beta {e}_2^2\right)\\ {}\kern4.25em -2\gamma \left[\alpha \left(1-2\alpha \right){d}_1{e}_1+\beta \left(1-2\beta \right){d}_2{e}_2-2\alpha \beta \left({d}_1{e}_2-{e}_1{d}_2\right)\right]\Big\}\end{array} $$

This leads to a sample variance of the preference effect itself as

11 $$ \begin{array}{l}\operatorname{var}\left(\overset{\wedge }{\varDelta \pi \Big) \approx}\right[\operatorname{var}\left( T*\right)+\frac{T{*}^2}{m\alpha \beta}\left(\alpha +\beta -4\alpha \beta \right)\\ {}\kern3.75em -\frac{2 T*\gamma}{\alpha \beta}\left\{\alpha \left(1-2\alpha \right){d}_1-\beta \left(1-2\beta \right){d}_2+\alpha \beta \left[\left(1-4\alpha \right){e}_1-\left(1-4\beta \right){e}_2\right]\right\}\Big]/{\left[2\alpha \beta m\right]}^2\end{array} $$

Similar derivations are possible for testing and estimating the second contrasts *Δν*′ and *Δπ*′; for brevity these are not shown here, but some simplified results are given below.

The results derived thus far are completely general in allowing for different variances in the various study subgroups, and for sample variation in the preference distribution within the choice arm. Considerable simplification occurs if these conditions are relaxed. First, we may assume that the outcome variance is constant. Second, we may ignore sample variability in the preference distribution in the choice arm. We refer to the variances as unconditional when sample variability in preferences is taken into account (as in Eqs. 7, 8, 9, 10 and 11), and conditional when the preference distribution is taken as fixed.

Making both assumptions together, the variances of the first estimated selection and preference contrasts become equal:

12 $$ \operatorname{var}\Big(\overset{\wedge }{\varDelta \nu \Big)=}\operatorname{var}\left(\varDelta \widehat{\pi}\right)=\frac{\sigma^2}{4{\alpha}^2{\beta}^2 m}\left[{\left(1-\gamma \right)}^3+2\left({\alpha}^2+{\beta}^2\right)\left(\gamma +\frac{\theta}{1-\theta}\right)\right] $$

as do the variances of the second estimated contrasts:

13 $$ \begin{array}{l}\operatorname{var}\left(\varDelta {\widehat{\nu}}^{\prime}\right)=\operatorname{var}\left(\varDelta {\widehat{\pi}}^{\prime}\right)=\frac{\sigma^2}{16{\alpha}^2{\beta}^2\gamma m}\Big[\gamma \left(1-\gamma \right){\left(\alpha -\beta \right)}^2+2\left\{{\alpha}^2{\left(2\beta +\gamma \right)}^2+{\beta}^2{\left(2\alpha +\gamma \right)}^2\right\}\\ {}\kern22.25em +2\gamma \left({\alpha}^2+{\beta}^2\right)\left(\frac{\theta}{1-\theta}\right)\Big]\end{array} $$

(detailed derivations not shown). As shown later in our example, the unconditional and conditional variances may be numerically very similar. Thus, in many practical situations, it will be sufficient to use the simpler conditional results (12) and (13).

### Possible assumptions for responses in undecided participants

In the original development of the two-stage design [2], Rucker proposed that one should either assume that there were no undecided participants (*γ* = 0), or that these people were not subject to selection or preference effects (*μ*_*i*3_ = *μ*_*i*_ for *i* = 1, 2, respectively). Because the {*μ*_*i*3_} are estimable from undecided participants within the choice arm, one can test the null hypotheses H0: *μ*_13_ = *μ*_1_ and H0: *μ*_23_ = *μ*_2_ by comparing the outcomes of the undecideds to their similarly treated counterparts in the random arm. When testing selection and preference effects, these assumptions are equivalent to assuming that *e*_*1*_ = *e*_*2*_ = 0; however, these terms appear in expressions such as (7) and (9, 10 and 11), implying that ignoring them can bias the test statistics or confidence intervals of these parameters.

An alternative approach is to assume that *μ*_*ii*_ = *μ*_*i*3_ for *i* = 1, 2, or that the expected outcomes are the same in participants with or without a preference, for each treatment. This amounts to the so-called “exclusion restriction” whereby the expected outcome is determined only by the treatment actually received. Again these assumptions are testable in the two-stage design, using data from the choice arm.

Another strategy would be to exclude undecided participants from the analysis. This approach would make it appear (incorrectly) that all participants in the random arm have a preferred treatment.

One can show that expressions (4) and (5) give consistent estimators of the selection and preference effects even in the presence of indifferent participants. Conversely if either set of assumptions above is adopted, but is actually false, biased estimation will occur. The bias will be important unless the proportion of indifferent participants is small.

## Results

To illustrate these calculations, we will use data from a trial to compare alternative interventions for heavy menstrual bleeding [6, 7]. The treatment options were surgery or medical management without surgery. Women were randomised to a “conventional trial” subgroup (random arm) or to a “patient preference” subgroup (choice arm). In the choice arm, women were asked if they had a preference for one of the treatments, and if so, they could have it.

There were 227 women with outcome data available, of whom 130 (57%) were assigned to the choice arm. Within the choice arm, 19 elected to take medical treatment, and 21 decided to have surgery. The remaining 90 women (69%) had no preference, and were randomised (45 to each treatment). Thus while the rate of undecided participants was high, the two treatments were selected in somewhat similar numbers by women who were able to make a choice.

Table 2 summarises one principal study outcome, observed using a bleeding score (higher values represent poorer outcomes). There were 5 women in the choice arm and 41 in the random arm who refused to participate after entering the study. The disparity in these numbers has the potential to create biased comparisons among the women who did agree to participate, but unfortunately outcomes are unavailable for the refusers. The lower refusal rate in the choice arm is in the expected direction, because some participants will be attracted by possibly being able to choose their own treatment.

**Table 2:** Summary of results from a two-stage randomised trial of medical vs. surgical treatment

Actual treatment		Choice arm (Total sample size = m)			Random arm (Total sample size = n)
		Chose medical	Chose surgical	Undecided (and randomised)	
Medical	Sample size	19		45	49
	Mean	16.6		18.4	17.2
	SD	8.7		10.7	5.2
Surgical	Sample size		21	45	48
	Mean		5.9	4.3	5.1
	SD		7.2	5.2	7.7

The investigators noted some baseline similarities between those choosing or being randomised to treatment, and they therefore aggregated the results from the randomised women in the random and choice arms, and reported outcomes only according to the actual treatment. In this paper we are able to use more detailed data on preferences within the choice arm (kindly provided by the investigators), allowing us to structure the results in the format of our Tables 1 and 2.

The direct effect of treatment (estimated from the random arm), was 12.10, with a standard error of 1.54 based on a standard deviation pooled over all the data. The 95% confidence interval was (9.1, 15.1), indicating a significant treatment effect in the direction of better outcomes for women having surgery.

The estimated first selection effect contrast $\varDelta \widehat{\nu}$ was 3.03; using the unconditional approach and allowing for different subgroup variances, with standard error 6.72. Testing this effect gives *T* = 18.6 (SD = 40.8); *z* = 0.46 (*p* = 0.65, 2-sided test). The conditional standard error of $\varDelta \widehat{\nu}$ when the outcome variance was taken as constant across study groups was 6.64, only fractionally smaller than its unconditional counterpart. For simplicity, conditional standard errors were used in all further analyses.

The second selection contrast $\varDelta {\widehat{\nu}}^{\prime }$ was 0.57, with conditional standard error 3.62. While there is no strong evidence of selection effects, the first contrast indicates better outcomes for women who would select surgery (treatment B) if allowed to do so, this being in addition to the direct benefit of surgery.

The two estimated preference effects are $\varDelta \widehat{\pi}$ = 0.93 and $\varDelta \widehat{\pi^{\prime }}$ = − 0.49 (standard error = 6.64 for both). The first contrast, being positive (although not significant), indicates that the medical versus surgical effect is greater for women who preferred medical treatment.

We can also empirically test the alternative assumptions for the outcomes among the undecideds. First, using Rucker’s approach [2], we can compare the outcomes for women on medical treatment, between the undecided participants and those in the random arm: this gives a mean difference of 18.40–17.20 = 1.20 (*z* = 0.77, *p* = 0.44). Similar analysis of the corresponding groups of women on surgery gives *z* = − 0.51 (*p* = 0.61). Second, an assessment of the “exclusion restriction” assumption leads to similar findings, with only small differences in outcomes between women who chose their treatment in the choice arm, or were assigned to it in the random arm. Hence, in this example, there is no compelling evidence of systematically different outcomes for undecided women. However, we feel it is still advantageous to allow for the existence of undecided participants, in order to avoid bias. As noted earlier, we do not recommend simply ignoring the data from undecided participants.

## Discussion

We have extended the methodology for two-stage randomised trials to allow for participants who do not have a preferred treatment. As noted earlier, the two-stage design has been used in a variety of settings, and in some cases the selection and preference effects have been important, even when lacking a large treatment effect. From the perspective of shared decision-making concerning treatment options, it would be important to know about these effects.

Investigators who consider adopting the two-stage trial design would presumably hope that most participants have a preferred treatment. For instance, trials using patient decision aids try to improve patient understanding of the treatment options and reduce the number of patients who are uncertain about their treatment, to help them make an informed choice reflecting their own values [19]. For patients who are unable or unwilling to choose, the decision will likely revert to the clinician proposing a treatment, and obtaining the tacit consent of the patient.

Despite the complication of undecided participants, the two-stage design remains attractive to people who do have a preferred treatment, and it still provides important information that is unobtainable from a conventional design. Preference effects may enhance the direct effect of treatment (as in our example), or act in the opposite direction. One may also compare the responses of decided vs. undecided individuals, as this may also be predictive of better or worse outcomes; these comparisons are accessible through our defined second contrasts of the selection and preference effects. Depending on the context, it seems better to allow study participants the option of declaring no preference, rather than pressuring them into a “forced” choice of treatment; in this scenario, “no preference” is a valid observation, and not “missing”. However, the question of undecided patients may not arise in some circumstances, for instance where one treatment is usual care (e.g., [4, 5]).

We remarked earlier that it is inappropriate to simply ignore undecided participants in the choice arm, because of the potential for bias. Interestingly, in our example study [6, 7], the investigators elected to include the undecided participants, but they aggregated them with similarly treated participants in the random arm. Because all patients in this analysis are randomised, the estimated treatment effect is unbiased, but it lacks generalizability because participants with a declared preference are excluded.

Despite the advantages of the two-stage design, it must be recognised that the existence of undecided or indifferent patients may reduce its appeal. First, if a large proportion of participants cannot decide on treatment, estimating all the effects of interest (treatment, selection and preference effects) becomes less precise. Second, the presence of undecided participants leads to greater analytic complexity. Finally, the burden of explaining the study to participants may become unattractive if relatively few people will actually be able to make a treatment choice. However, the investigators in our example found that using this design “did not affect recruitment” compared to conventional approaches [6, 7]. Nevertheless, it should be noted that despite this encouraging finding, outcomes may still differ between participants in the random arm and the subgroup who are randomised within the choice arm.

In our example [6, 7], there was a high proportion of undecided participants, with less than one third of the patients in the choice arm declaring a preference. In this study, the information sheet for women in the choice arm contained only two additional sentences (“*We do realise that some women may have a preference for one of the treatments. If you feel strongly that you want one treatment in particular, then please tell us*”) compared to the information provided in the random arm. The mention of “strong” preferences may have deterred some women from choosing a treatment for which they had only a weaker preference. Researchers carrying out preference trials should consider carefully the methods they use to elicit preferences and their impact on the preferences that participants feel able to express.

Participants with no preference were also documented in another two-stage design [9], a trial comparing medication and cognitive behavioural therapy for the treatment of depression. Preferences were recorded on a 5-point scale, with participants (19%) who chose the middle value being defined as having “no clear treatment preference” or “indecisive”. In other examples of the two-stage design [8, 16–18, 20], no undecided participants were reported, but it is unclear if such individuals were simply ignored, or if initially undecided persons were somehow persuaded to make a definitive choice before continuing into the study.

Further work is required to extend our earlier results [3] on optimising study efficiency while allowing for undecided participants. Optima here will depend on the proportion of participants who can decide on a treatment, as well as factors such as the preference rates for the two treatments among the decided individuals. The preference distribution will also affect the power to detect selection and preference effects [21].

We limited our attention to trials with equal numbers of participants being assigned to each treatment group in the random arm. Another research topic of future interest will therefore be to extend our methods to allow for unequal treatment group sizes. Unequal assignments might be desirable for ethical reasons, for instance if there is a large majority preference for one treatment, or if one treatment has a larger response variance. In such cases, the investigators might wish to to improve study efficiency, by striving for overall balance in the trial, for example. We have discussed the ethics of unequal randomisation in somewhat more detail elsewhere [1].

We adopted a model in which preferences were assumed to have fixed effects. This was to conform to Rucker’s original development, and indeed to our own earlier work in this area, which we have extended here. A further interesting extension would be to allow for preferences (and, potentially, the treatment) to have random effects. We note that all our estimates and tests are based on large sample normal approximations, and further work would be needed to consider small sample situations. One could, for instance, replace our z-statistics with t-statistics. Our previous work on sample size and power for the two-stage design [21] showed that particularly small studies will be inadequate to reliably estimate the parameters of interest. Rucker’s investigation of empirical type I error rates showed excellent adherence to nominal type I error rates with as few as 20 patients in each of the random and choice arms [2]. These results suggest that the large sample approximations described here will be adequate for most practical problems.

A reviewer pointed out that participants in the random arm may be randomised to a treatment that they do not like, and this in turn may influence outcomes. This is a valid point, but one that also affects a number of other designs, including standard parallel group trials, in exactly the same way. It has previously been noted that parallel group trials involve arms that may not be comparable after randomisation, because some patients will be pleased to get their preferred treatment, while others will be disappointed (this assuming that participants are not blinded) [22]. This phenomenon has been termed “reluctant acquiescence” [23], and it may in turn affect the estimated treatment effect. To the extent this is a problem for the two-stage design, exactly the same problem exists in parallel group designs, in terms of the impact on the estimated treatment effect. However, note that because the estimates of the selection and preference effects in a two-stage design are based partly on the data from the random arm, there may also be subsidiary implications for those estimates as well.

There are related issues in a variety of other preference-based designs, as we have reviewed elsewhere [1]. For example, there are challenges in how to present information about treatments and then eliciting preferences reliably, and one must recognise that there are potential differences between what are stated as the preferences for treatment in a general sense, and actual treatments chosen when participants are allowed to choose.

As we have reviewed elsewhere [1], randomised trial designs can allow for patient preference effects in various ways. There is also some literature on the impact of clinician preferences for treatments, and the associated “expertise bias” [24, 25]. The various designs that accommodate patient or clinician preferences are differentiated by whose preference is ascertained, how and when they are measured, and if treatment assignments take those preferences into account. Among these designs, the two-stage approach is unique in being able to estimate selection and preference effects in addition to the direct treatment effects [26, 27]. It remains a candidate design even when the complication of indifferent participants arises. The results in this paper have shown how the information from such participants can be used in the analysis for two-stage trials, to further elucidate the impact of treatment preferences on study outcomes.

## Conclusions

Treatment preferences among clinical trial participants are potentially important determinants of study outcomes, and we have demonstrated how data from two-stage randomised trials can be analysed to estimate such effects. We have extended our earlier work to now allow for the possibility of participants who are indifferent or undecided about a preferred treatment, and we discussed alternative possible assumptions about their response patterns, compared to participants with a declared treatment preference. We also have shown how to obtain unbiased estimates and tests of the preference effects in the trial as a whole. Despite the presence of indifferent or undecided participants, the two stage design is nevertheless an attractive option for the examination of preference effects, a feature which is not possible with conventional trial designs.

## Additional file

Additional file 1: The Appendix provides a more detailed outline of the derivations of the test statistics and variances associated with estimates of selection and preference effects described in this paper. (DOCX 64 kb)

## Abbreviations

SD: Standard deviation
