# Correction to: Estimation of treatment preference effects in clinical trials when some participants are indifferent to treatment choice

**DOI:** 10.1186/s12874-020-00967-3

**Published:** 2020-04-14

**Authors:** Stephen D. Walter, Robin M. Turner, Petra Macaskill, Kirsten J. McCaffery, Les Irwig

**Affiliations:** 1 Department of Clinical Epidemiology and Biostatistics, McMaster University, CRL 233, Hamilton, ON L8N 3Z5 Canada; 2 School of Public Health and Community Medicine, University of New South Wales, Sydney, NSW 2052 Australia; 3 Screening and Test Evaluation Program, Sydney School of Public Health, Sydney Medical School, University of Sydney, Sydney, NSW 2006 Australia

**Correction to: BMC Med Res Methodol**

**https://doi.org/10.1186/s12874-017-0304-x**

In the original publication of this article [1], there is a small calculational error in the illustrative example. The estimated second preference contrast Δπ' should be -3.23, not -0.49 as shown. All the other numerical results in the example are shown correctly. This change does not materially affect the substantive interpretation of the analysis.
