# Supplementary material for: Estimation of treatment preference effects in clinical trials when some participants are indifferent to treatment choice
Source: BMC Med Res Methodol. 2017 Feb 20;17:29. doi: 10.1186/s12874-017-0304-x (PMC5319089; doi:10.1186/s12874-017-0304-x)
Supplement: Additional file 1: — The Appendix provides a more detailed outline of the derivations of the test statistics and variances associated with estimates of selection and preference effects described in this paper. (DOCX 64 kb) [file 12874_2017_304_MOESM1_ESM.docx]

**APPENDIX: ADDITIONAL TECHNICAL DETAILS**

In this Appendix we provide an outline of the derivations of the test statistics and variances associated with estimates of selection and preference effects.

First, we consider the test statistic *T* used to evaluate the null hypothesis of no selection effect, as in equation (6). Given that the test statistic is $T=\left(z_{1}-z_{2}\right)-\hat{\gamma}(w_{1}-w_{2})$, we may write its variance as follows:

(A1)

Then we may consider the three components of (A1) in turn. For the first component, $\mathrm{var}\left(z_{1}-z_{2}\right)$, recalling that and , we require expressions for four variance terms such as $\mathrm{var}(m_{1}\bar{X}_{1})$ and $\mathrm{var}(m_{2}\bar{Y}_{2})$, and six covariance terms such as and . Noting that the sample means are mutually independent, and that the sample sizes (*m_1_, m_2_, m_3_*) have a multinomial distribution with associated probabilities (*α, β, γ*), we may use selected results from our earlier work [3] to establish that

$\mathrm{var}(z_{1}-z_{2})\approx m\left[\alpha d_{1}^{2}+\beta d_{2}^{2}-(\alpha d_{1}-\beta d_{2})^{2}+\alpha\left(\sigma_{11}^{2}+\frac{2\alpha\theta}{1-\theta}\sigma_{1}^{2}\right)+\beta\left(\sigma_{22}^{2}+\frac{2\beta\theta}{1-\theta}\sigma_{2}^{2}\right)\right]$ (A2)

where $d_{1}=\mu_{11}-\mu_{1}$*, $d_{2}=\mu_{22}-\mu_{2}$ ,* $\theta=m/N$ and assuming *n*_1_ = *n*_2_ [cf. 3, equation A2].

The second component of (A1) may be written in terms of the observable sample means as

(A3)

Because the sample means are independent of the multinomially distributed subgroup sample sizes, the first term is the variance of an independent product [26]: hence

(A4)

The sample means $\bar{X}_{1}$and $\bar{V}_{1}$are based on sample sizes *mα* and *mγ/2*, leading to their variances and the variance of the difference in the usual way. Moments and joint moments of the sample frequencies (*m_1_, m_2_, m_3_*) can be obtained using the joint factorial moments of the multinomial distribution [ref. 26, page 33, eqn. 35.5], specifically,

$E(m_{1}^{(r1)}m_{2}^{(r2)}m_{3}^{(r3)})=m^{(r1+r2+r3)}\alpha^{r1}\beta^{r2}\gamma^{r3}$ (A5)

where *m = m_1_+ m_2_ + m_3_*, and where, *m*(*^k^*) indicates the *k*^th^ factorial moment; for example, *m^(3)^= m*(*m* – 1)(*m* – 2). Hence, in particular, $E(m_{1}m_{3})=m(m-1)\alpha\gamma\approx m^{2}\alpha\gamma$ after taking the leading terms, which are of O(*m^2^*).

Another application of (A5) gives$E(m_{1}^{2}m_{3}^{2})=m(m-1)\alpha\gamma[(m-2)(m-3)\alpha\gamma+(m-2)(\alpha+\gamma)+1]$, and hence, after cancelling terms of O(*m^4^*) and collecting the remaining leading terms of O(*m^3^*), we have $\mathrm{var}(m_{1}m_{3})=E(m_{1}^{2}m_{3}^{2})-[E(m_{1}m_{3})]^{2}\approx m^{3}\alpha\gamma(\alpha+\gamma-4\alpha\gamma)$. Putting these results together gives

(A6)

where $e_{1}=\mu_{11}-\mu_{13}$; we may also obtain a symmetrically equivalent expression for .

Again, because the sample means are independent, the third term in (A3) can be written:

(A7)

and from the general result (A5) we have that

$\mathrm{cov}(m_{1}m_{3},m_{2}m_{3})\approx\alpha\beta\gamma m^{3}(1-4\gamma)$ (A8)

Combining (A4), (A6) - (A8) gives the required result for $\mathrm{var}[\hat{\gamma}(w_{1}-w_{2})]$as in (A3).

The third component of (A1) is

$\mathrm{cov}[\left(z_{1}-z_{2}\right),\hat{\gamma}(w_{1}-w_{2})]=E\left[\frac{m_{3}}{m}\left(z_{1}-z_{2}\right)(w_{1}-w_{2})\right]-E\left(z_{1}-z_{2}\right).E\left[\frac{m_{3}}{m}(w_{1}-w_{2})\right]$ (A9)

which can be approached using similar techniques. Specific multinomial moments required from (A5) include $E(m_{1}^{2}m_{3})=m(m-1)\alpha\gamma[(m-2)\alpha+1]$, $E(m_{1}m_{2}m_{3})=m(m-1)(m-2)\alpha\beta\gamma$, and symmetrically equivalent terms. Terms from (A9) which are O(m^2^) cancel, leaving leading terms of O(m). When these components are combined, we obtain

(A10)

where $e_{2}=\mu_{22}-\mu_{23}$*.* Finally, combining results (A2), (A6) and (A10) gives the expression for var(*T*) as in equation (7) of the main text. This can be used for testing the significance of the selection effect.

Some further development is needed to establish a variance for the selection effect. Because *,* by applying the delta method we can derive an approximate variance:

(A11).

as shown in (8). The first component of (A11), var(*T*), has already been derived, as above, and is shown as equation (7). The second component,$\mathrm{var}(\hat{\alpha}\hat{\beta}$, can be obtained by again using the multinomial moments from (A5), specifically:

The third component,$\mathrm{cov}(T,\hat{\alpha}\hat{\beta}$, can be approached similarly, involving terms such as and , as well as variances and covariances for the elements of *T*, and for their products with the multinomial frequencies.

Derivations for the variance of the test statistic *T** for the preference effect (equation 10), and for the variance of the estimated preference effect (equation 11) can be obtained using methods similar to those shown above, apart from some changes of sign for component terms in the elements of these expressions.
